# Soft Tissue Tumor Without Neurofibromatosis Type 1 With Histopathological Suspicion of Atypical Neurofibromatous Neoplasm of Uncertain Biological Potential (ANNUBP)

**DOI:** 10.7759/cureus.38187

**Published:** 2023-04-27

**Authors:** Hiroyuki Tsuchie, Hiroyuki Nagasawa, Hiroshi Nanjyo, Naohisa Miyakoshi

**Affiliations:** 1 Orthopedic Surgery, Akita University Graduate School of Medicine, Akita, JPN; 2 Pathology, Akita University Graduate School of Medicine, Akita, JPN

**Keywords:** positron-emission tomography, older adult, atypical neurofibromatous neoplasm, neurofibromatosis type 1, soft tissue tumor

## Abstract

Atypical neurofibromatous neoplasm of uncertain biological potential (ANNUBP), defined as a borderline lesion that is difficult to distinguish whether benign or malignant, is one of the intermediate stages to malignant peripheral nerve sheath tumor, a peripheral nerve-derived malignant tumor that develops from nerve sheath cells. Because ANNUBP is a new concept, only a few cases have been reported, all in patients with neurofibromatosis type 1 (NF-1).An 88-year-old woman presented with a mass on the left upper arm persisting for one year. Magnetic resonance imaging showed a large tumor spreading between the humerus and biceps muscle, which was diagnosed as undifferentiated pleomorphic sarcoma by needle biopsy. Extensive tumor resection was performed, including partial cortical bone resection of the humerus. Based on the histological features, although the patient did not have NF-1, the tumor was strongly suspected to be ANNUBP. As malignant peripheral nerve sheath tumors have been sporadically reported in patients without NF-1, it is feasible that ANNUBP could also occur in patients without NF-1.

## Introduction

Occasional malignant transformation of a neurofibroma into malignant peripheral nerve sheath tumor (MPNST) is known to occur in patients with neurofibromatosis type 1 (NF1), and progresses in multiple stages. Because the histological diagnostic criteria for MPNST were unclear, a consensus meeting was held in 2016 by experts in the pathology of soft tissue and peripheral nerve tumors, and certain diagnostic criteria were established [1]. Currently, atypical neurofibromatous neoplasm of uncertain biological potential (ANNUBP) is defined as a borderline lesion that is difficult to distinguish between benign and malignant, and is one of the intermediate stages to MPNST. Because ANNUBP is a relatively new concept, very few cases have been reported, all in patients with NF-1 [2-4].

Here, we describe an extremely rare case of an upper arm soft tissue tumor in an older adult without NF-1, which was histopathologically strongly suggestive of ANNUBP, and we conducted a review of the available literature.

## Case presentation

**Figure 1:**
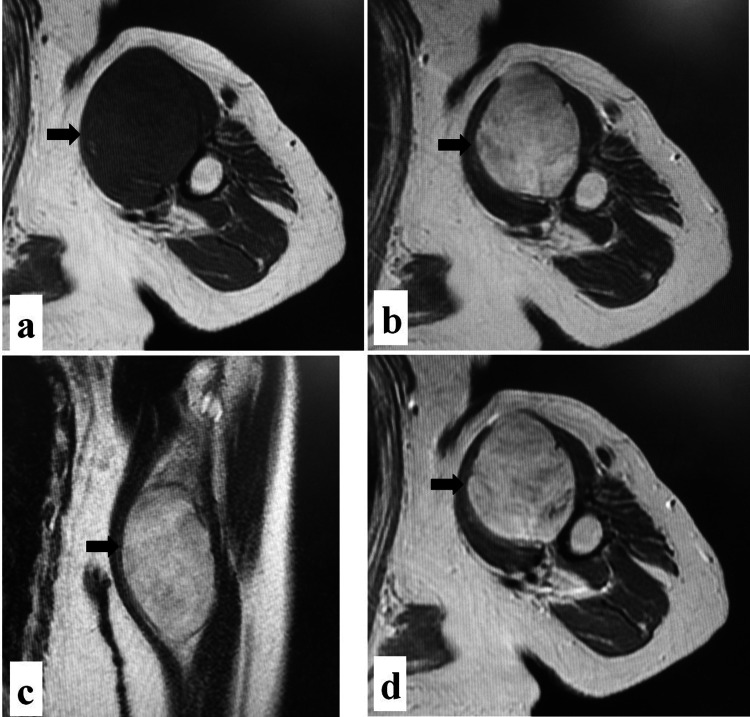
Magnetic resonance images of the left upper arm. (a) Axial T1-weighted, (b) axial T2-weighted, (c) coronal T2-weighted, and (d) axial T1-weighted contrast-enhanced images showing a tumor with iso signal intensity on T1-weighted, mild hyperintensity on T2-weighted, and whole-tumor enhancement on contrast-enhanced images (arrows).

**Figure 2:**
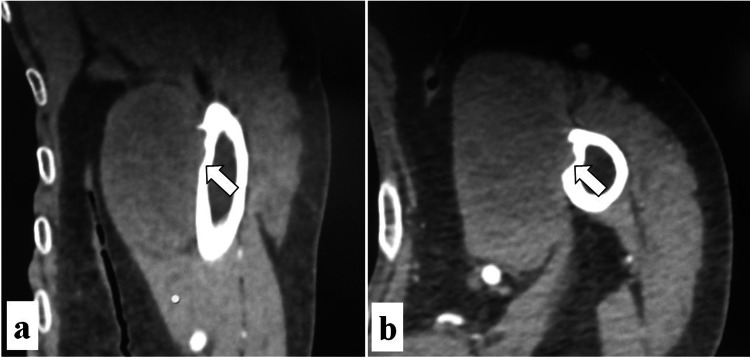
Computed tomography images of the left upper arm. (a) Coronal and (b) axial images showing the tumor partially encroaching into the cortical bone on the medial side of the humerus (white arrows).

**Figure 3:**
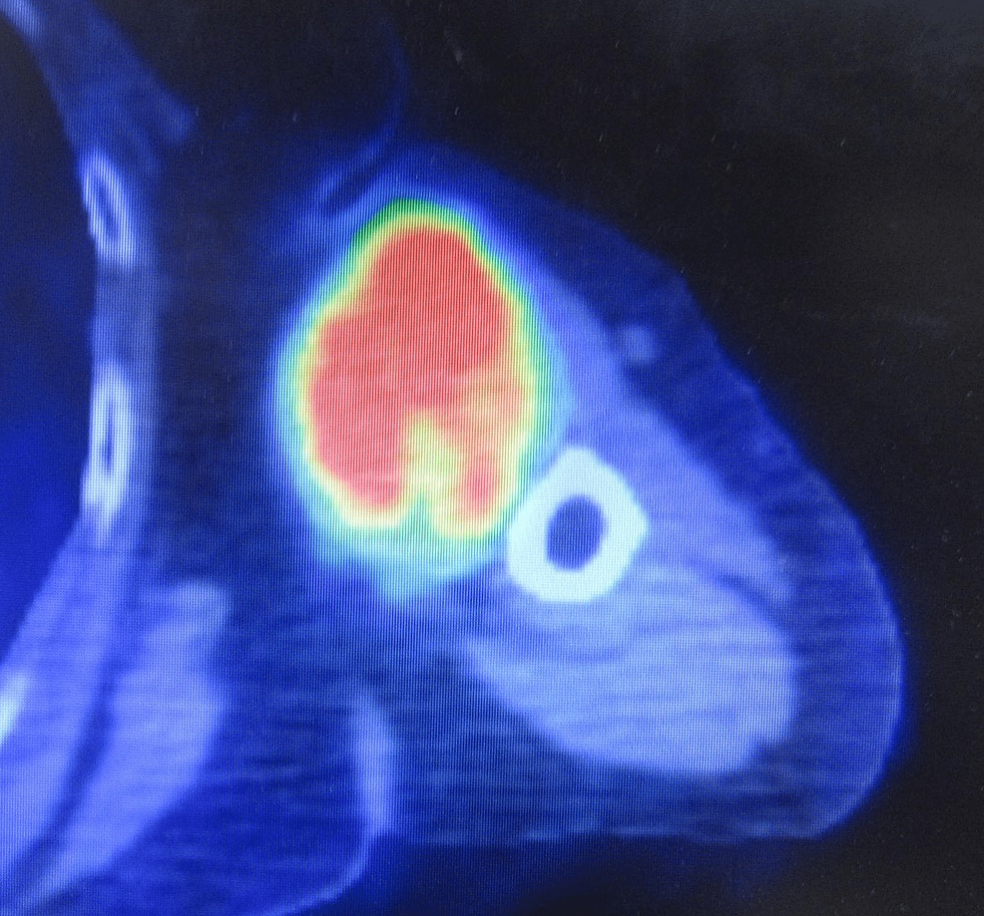
Positron-emission tomography/computed tomography image (axial view) demonstrating tracer uptake in the upper arm tumor (SUVmax 8.71).

An 88-year-old woman undergoing treatment for hypertension presented at our outpatient clinic due to a mass on the left upper arm persisting for one year. Physical examination revealed a hard lump on the anterior aspect of the left upper arm. Magnetic resonance imaging showed a large tumor (maximum diameter: 75 mm) spreading between the humerus and biceps muscle. The tumor showed iso signal intensity on T1-weighted images (Figure 1a), mild hyperintensity on T2-weighted images (Figure 1b-1c), and whole-tumor enhancement on contrast-enhanced images (Figure 1d). Computed tomography (CT) showed the tumor partially encroaching into the cortical bone on the medial side of the humerus (Figure 2). Soft tissue sarcoma was suspected and needle biopsy was performed, which returned the diagnosis of undifferentiated pleomorphic sarcoma. Positron-emission tomography (PET)/CT demonstrated a maximum standardized uptake value (SUVmax) of 8.71 for the tumor, and no abnormal uptake was observed at other sites (Figure 3).

**Figure 4:**
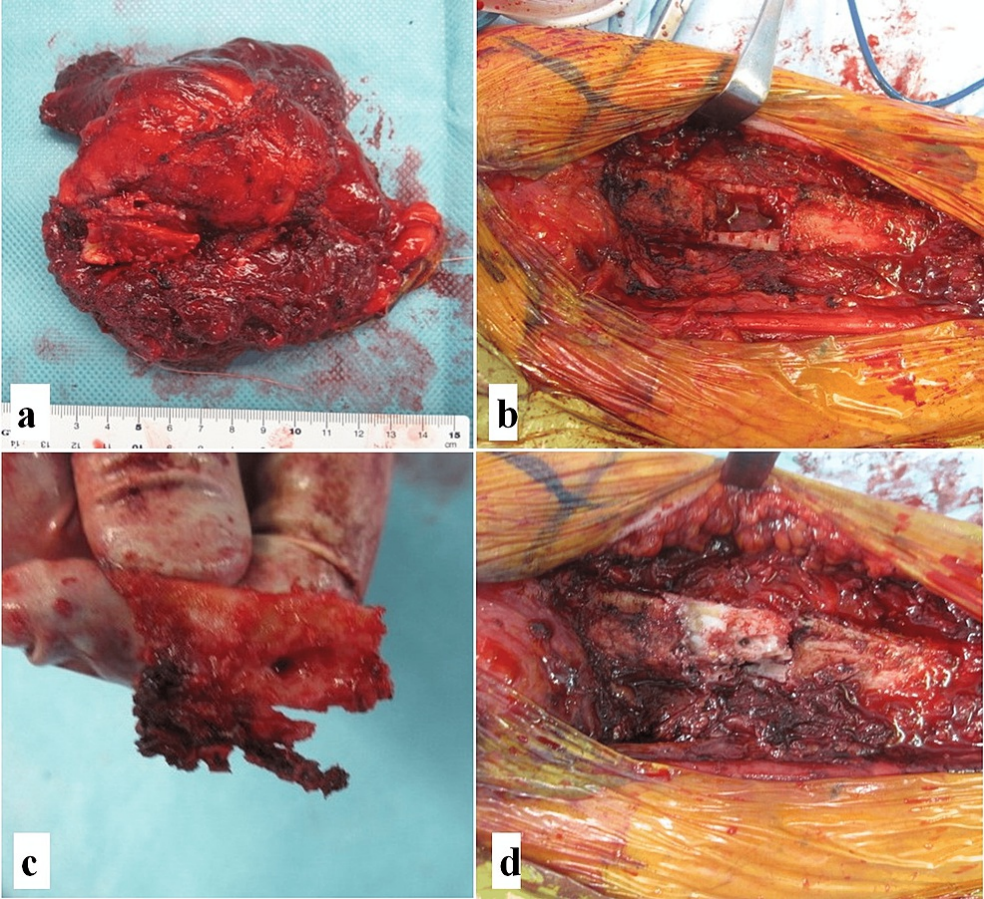
Intraoperative photographs. Extensive tumor resection was performed and part of the cortical bone of the humerus was resected with the tumor remaining as one mass (a, b). Part of the tumor had penetrated the humerus, causing a partial depression of the cortical bone (c). After the cortical bone was immersed in hot water, it was put back in place (d).

**Figure 5:**
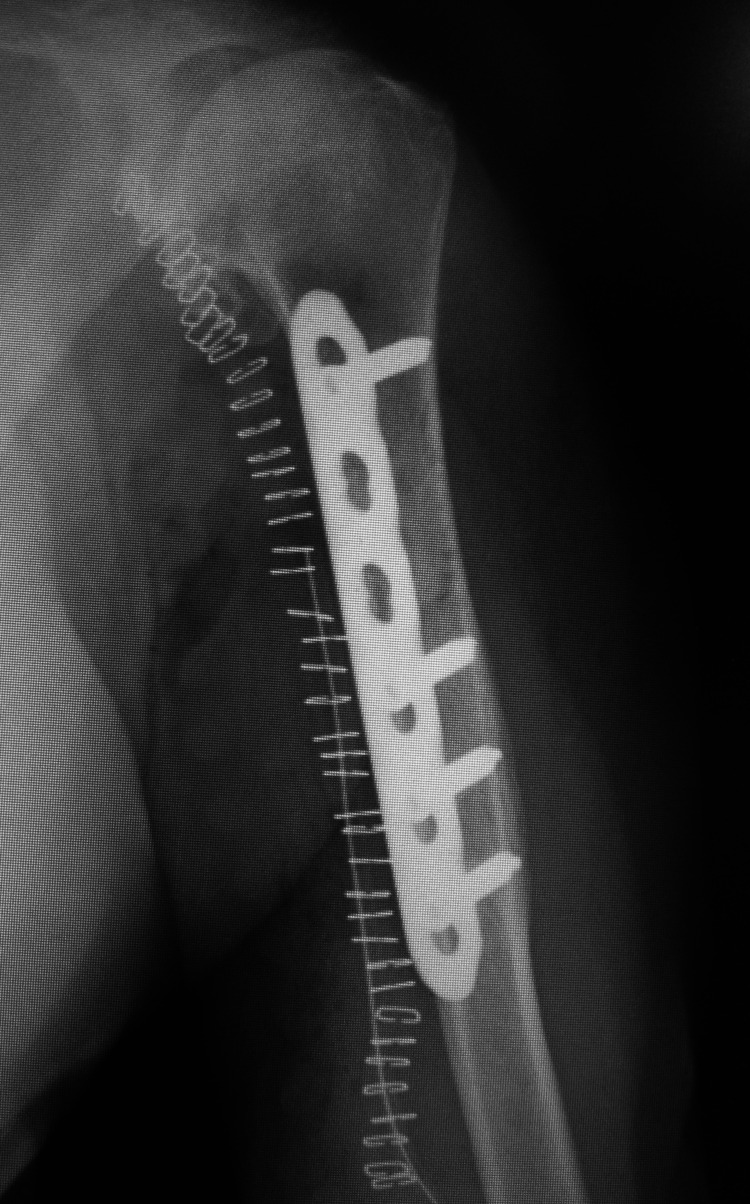
Postoperative antero-posterior radiograph of the left upper arm showing the fragment of cortical bone fixed with a plate.

**Figure 6:**
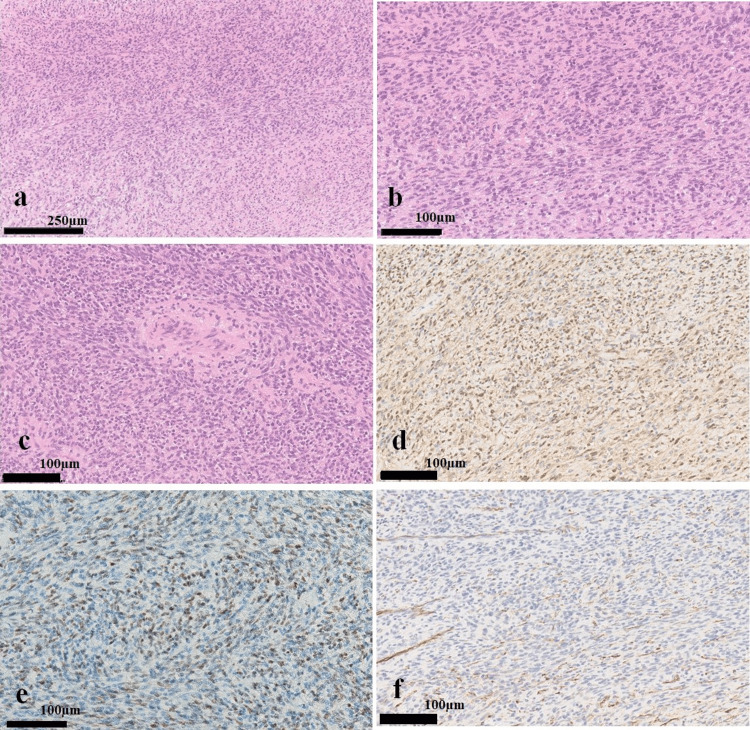
Histopathological findings. Photomicrographs showing spindle-shaped cells growing intricately in fascicles with perivascular hyalinization, increased cell density, and nuclear atypia, with approximately 3 mitosis observed per 50 high power field (hematoxylin and eosin staining, magnification ×100 and ×200) (a, b, c). Immunohistochemical staining confirmed diffuse positivity for S-100 and SOX10 (magnification ×200) (d,e) and partial positivity for CD34 (magnification ×200) (f).

Extensive tumor resection was performed, including partial cortical bone resection on the medial side of the humerus (Figure 4). After the cortical bone was immersed in hot water, it was put back in place and fixed with a plate (Figure 5). Histopathological analysis revealed spindle-shaped cells growing intricately in fascicles with perivascular hyalinization, increased cell density, and nuclear atypia, with approximately 3 mitosis/50 high power field (HPF) (Figure 6a-6c). Immunostaining confirmed diffuse cytoplasmic positivity for S-100 and SOX10 (Figure 6d-6e), and partial positivity for CD34 (Figure 6f). The Ki-67 labeling index was 8.3%. Based on these histological features, although the patient had no history of, or neurofibroma suggestive of, NF-1, the tumor in her upper arm was strongly suspected to be ANNUBP.

The postoperative course was uneventful, and there has been no local recurrence up to the most recent follow-up, two years and nine months postoperatively.

## Discussion

Based on the histological characteristics, the stages of malignant transformation of a neurofibroma into MPNST are defined as neurofibroma with atypia, cellular neurofibroma, ANNUBP, low-grade MPNST, and high-grade MPNST [1]. ANNUBP is a Schwann cell-derived tumor that meets at least two of the following four criteria: nuclear atypia, loss of neurofibroma architecture, hypercellularity, mitotic index > 1/50 HPF and <3/10 HPF. In our case, three of the four criteria were met-nuclear atypia, hypercellularity, and mitotic index > 1/50 HPF and < 3/10 HPF-leading to the diagnosis of ANNUBP. Because the concept of this disease was originally proposed in patients with NF-1, it is difficult to judge whether ANNUBP is applicable to patients without NF-1. All previously reported cases occurred in patients with NF-1. However, MPNST is a peripheral nerve-derived malignant tumor that differentiates into nerve sheath cells, and there are sporadic cases in patients without NF-1 [5]. Therefore, in theory, it would not be contradictory for ANNUBP to occur in patients without NF-1.

As only five cases have been reported, of which only two are detailed and include images, little is known about the clinical features of ANNUBP at present [2-4]. Compared to the average age of 21.2 years (9-41 years) in the past five cases, our patient was significantly older (88 years). Given that all past cases were reported in patients with NF-1, the absence of NF-1 in our patient may be related to the onset at old age. There are no reports that have fully examined the resection margins, and in many cases, marginal resection was performed. No local recurrence was reported in any case, but the average follow-up period in previous reports was as short as 1.36 years (1-2.3 years). In our case, the follow-up period was 2.5 years, which is slightly longer than the previously reported, but insufficient to reflect the long-term progress. Comprehensive long-term follow-up is necessary for future evaluation of resection margins and local recurrence.

The usefulness of PET/CT as diagnostic imaging in ANNUBP has been previously reported [3]. The average SUVmax in the four patients who underwent PET/CT was 7.34 (6.3-8.6), while in our case, it was slightly higher at 8.71, but close to the previously reported. Some authors have suggested that the SUVmax cutoff value for discriminating MPNST from neurofibroma in NF1 cases is between 3.5 and 7.48 [6,7]. Tumors with SUVmax of 6 or higher in patients with NF1 may have progressed to ANNUBP or MPNST, and histological evaluation is considered necessary [7].

## Conclusions

We presented an extremely rare case of an upper arm soft tissue tumor strongly suggestive of ANNUBP in an older adult without NF-1. As MPNST have been sporadically reported in patients without NF-1, it is feasible that ANNUBP could also occur in patients without NF-1. ANNUBP is still a new disease concept and clinical information is scarce; thus, more reports are needed in the future.
